# Association of TyG index with sepsis incidence and mortality: a prospective study with diabetes stratification

**DOI:** 10.3389/fendo.2026.1834832

**Published:** 2026-05-28

**Authors:** Shuying You, Zhenhua Xing, Jiaqing Hu

**Affiliations:** 1 The Second People’s Hospital of Hunan Province (Brain Hospital of Hunan Province), Changsha, China; 2 Department of Emergency Medicine, Second Xiangya Hospital, Central South University, Changsha, Hunan, China

**Keywords:** cohort, diabetes, sepsis, sepsis-related mortality, the triglyceride-glucose index

## Abstract

**Background:**

The triglyceride-glucose (TyG) index, a validated surrogate marker of insulin resistance, has been widely investigated for its associations with cardiovascular outcomes in both diabetic and non-diabetic populations. However, its potential role in sepsis development remains largely unexplored. Moreover, whether the relationship between the TyG index and sepsis risk differs according to diabetes status has not been systematically examined.

**Methods and results:**

In this prospective cohort study, we analyzed 428,207 participants from the UK Biobank who had baseline TyG index measurements and no prior history of cardiovascular disease. Associations between the TyG index and sepsis incidence and sepsis-related mortality were assessed using Cox proportional hazards models, with stratification by diabetes status to evaluate potential effect modification. Over a median follow-up of 13.04 years, 12,410 sepsis events and 6,291 sepsis-related deaths were documented. Among individuals with diabetes, the relationship between the TyG index and both sepsis incidence and mortality was linear, whereas a U-shaped association was observed in non-diabetic participants. After adjustment for potential confounders, each 1-unit increase in the TyG index was associated with an 18% higher risk of sepsis (hazard ratio [HR]: 1.18; 95% confidence interval [CI]: 1.10–1.27) and a 16% higher risk of sepsis-related mortality (HR: 1.16; 95% CI: 1.05–1.27) in the diabetic subgroup. In contrast, no significant associations were observed among non-diabetic participants for either sepsis incidence (HR: 1.02; 95% CI: 0.98–1.06) or sepsis-related mortality (HR: 0.99; 95% CI: 0.94–1.05). Importantly, inclusion of the TyG index improved the predictive performance of risk models for both outcomes in individuals with diabetes.

**Conclusion:**

These findings demonstrate a diabetes-status-dependent association between the TyG index and sepsis outcomes. Elevated TyG levels were linearly and independently associated with increased risks of sepsis and sepsis-related mortality in diabetic individuals, whereas no significant associations were observed in non-diabetic participants. This suggests that the TyG index may serve as a clinically useful biomarker for sepsis risk stratification in patients with diabetes, warranting further validation in external cohorts.

## Background

Sepsis, defined as a life - threatening condition characterized by acute organ dysfunction resulting from a dysregulated host response to infection, poses a substantial global health challenge. Globally, it is approximated that there are 48.9 million cases of sepsis and 11 million associated deaths each year. In the United States, sepsis accounts for over one - third of in - hospital mortalities (1). In 2017, the cost related to sepsis surpassed $38 billion (2). As a result, sepsis not only represents the leading cause of in - hospital death but also the most costly reason for hospitalization, highlighting the urgent need to identify risk factors to mitigate its impact.

The triglyceride-glucose (TyG) index, a well-established marker of insulin resistance, has been implicated in the pathogenesis of obesity, nonalcoholic fatty liver disease, hypertension, autonomic neuropathy, and cardiovascular diseases (CVD) (3). Emerging evidence highlights its potential role in sepsis outcomes, although reported associations remain inconsistent: Cao et al. reported a negative correlation with clinical outcomes (4), Lou et al. and Zheng et al. observed positive associations (5, 6), and Fang et al. identified a U-shaped relationship with sepsis prognosis (7). The mechanistic basis for these divergent findings remains unclear; critically, while existing studies have focused primarily on sepsis outcomes (e.g., mortality and prognosis), whether the TyG index is associated with sepsis incidence—specifically the risk of developing sepsis—remains unknown. Notably, He et al. demonstrated diabetes status modifies the TyG-CVD association, showing a positive correlation in diabetic individuals but a U-shaped relationship in non-diabetics (8). Whether a similar diabetes-dependent modification exists for the TyG-sepsis association—particularly at the stage of sepsis incidence (i.e., susceptibility to initial sepsis development)—remains unexplored.

While existing studies have focused on TyG as a prognostic marker for sepsis outcomes (e.g., mortality, organ dysfunction) (6, 7), two critical gaps persist: 1) whether the TyG index represents a risk factor for sepsis incidence and could inform primary prevention strategies; and 2) whether diabetes status modifies this association at the disease initiation stage (not just during post-sepsis prognosis). To address the critical unmet need for understanding the causal role of the TyG index in sepsis pathogenesis—specifically whether it reflects a modifiable risk factor for sepsis incidence (not just prognosis)—and the potential modifying effect of diabetes status, we conducted a prospective analysis using data from the UK Biobank. Our objectives were twofold: 1) to characterize the dose-response associations between baseline TyG index and subsequent sepsis incidence and all-cause mortality in a large population-based cohort; and 2) to determine whether these associations are modified by diabetes status (diabetic vs. non-diabetic).

## Methods

### Study population

The UK Biobank is a prospective, population-based cohort comprising over 500,000 participants, enrolled from across the United Kingdom during 2006–2010. At baseline, standardized physical examinations, biospecimen collection, and detailed evaluations of demographic characteristics, lifestyle factors, and medical history were conducted through touchscreen questionnaires and nurse-administered interviews. Ethical approval was granted by the North-West Multi-Centre Research Ethics Committee (reference: 11/NW/0382), and all participants provided written informed consent. To safeguard privacy, all data were anonymized, with no identifiable information linked to research outputs. Participant involvement was restricted to data provision to ensure methodological impartiality; no contributions were made to study design, execution, or analysis. The full protocol and data specifications are available in version-controlled documentation (http://www.ukbiobank.ac.uk/researchers/).

### TyG index calculation and study outcomes

The TyG index was calculated using the established formula: TyG index = ln[fasting triglycerides (mg/dL) × fasting glucose (mg/dL)/2] (3). The study outcomes included incident sepsis and sepsis-related mortality, with sepsis cases identified from ICD-10-coded secondary care records (A02, A39, A40, A41) (9). Sepsis-related mortality was defined as death within 28 days post-sepsis diagnosis or critical care admission for sepsis (9). Follow-up spanned from enrollment to the earliest of first sepsis diagnosis, sepsis-related mortality, or March 31, 2022 (censoring date).

### Covariates

Baseline characteristics were systematically gathered through multiple methods, including questionnaires, interviews, and medical record reviews. Demographic variables were comprehensively captured, covering age, gender (male/female), race (White/Non-White), and educational attainment (college/university, below college level, or non-response). Lifestyle factors were also thoroughly assessed, with smoking status self-reported by participants and categorized as never smoker, current smoker, former smoker, or missing data. Alcohol consumption was classified into current drinker, former drinker, and non-drinker. Diabetes history was documented based on participant self-reports, and further validated by physician diagnoses or hospital records. Socio-economic status was evaluated using the Townsend Deprivation Index (TDI), which was calculated from national census data to identify regions with different levels of deprivation (10, 11). Dietary habits were quantitatively evaluated via a scoring system, which gauged compliance with essential dietary guidelines. These included consuming at least four tablespoons of vegetables (raw or cooked) daily, three servings of fruit per day, fish at least twice per week, and limiting processed meat intake to no more than twice per week. Scores ranged from 0 to 4 based on the number of criteria met (12). Physical health parameters, including body mass index (BMI), blood pressure [systolic (SBP) and diastolic (DBP)], and lipid profiles [low-density lipoprotein (LDL) and high-density lipoprotein (HDL)], were measured at baseline during the assessment center visit using standardized procedures.

### Statistical analysis

Categorical variables were analyzed using chi-square tests, whereas continuous variables were evaluated with either analysis of variance (ANOVA) or Mann–Whitney U-tests, contingent upon their distributional properties. To elucidate the associations between the TyG index and the incidence of sepsis or sepsis-related mortality, stratified by diabetes status, Cox proportional hazards regression models were implemented. Two regression models were developed: Model 1 was adjusted for age, sex, and race; Model 2 further incorporated additional covariates, including the TDI, MET levels, dietary score, tobacco and alcohol consumption, educational attainment, employment status, and BMI. To investigate potential nonlinear relationships between the TyG index and outcomes, stratified by diabetes status, restricted cubic splines with four knots were employed to model the dose–response associations. The relative importance of the TyG index in predicting sepsis and sepsis-related mortality was quantified by calculating the R² values derived from the Cox proportional hazards models adjusted for covariates in Model 2 (13). To evaluate the incremental predictive value of the TyG index beyond the covariates in Model 2, we calculated Harrell’s C-statistic, the Net Reclassification Improvement (NRI), and the Integrated Discrimination Improvement (IDI). All analyses were stratified by diabetes status to assess whether the improvement in model performance remained consistent across different metabolic subgroups (14). The proportional hazards assumptions were rigorously verified using Schoenfeld residuals, and all models satisfied these assumptions. Subgroup analyses were conducted across age categories (≤60 vs. >60 years), sex, and obesity status (BMI ≥ 30 kg/m² vs. BMI < 30 kg/m²). Five sensitivity analyses were performed: 1) exclusion of sepsis cases occurring within the first two years of follow-up; 2) right-censoring of data at age 70 years; 3) removal of individuals with missing data; and 4) additional adjustment for antihyperglycemic medications, LDL, HDL, SBP, CRP, DBP, and a history of chronic kidney disease (CKD); 5) use of the average TyG index for participants with available repeated measurement data to account for potential longitudinal changes in metabolic status. Competing risk models were applied to account for non-sepsis-related mortality, thereby mitigating potential biases that could confound the analysis of sepsis onset. All statistical tests were two-sided, with statistical significance set at P < 0.05. Analyses were conducted using Stata/MP 17.0 software (StataCorp LLC, College Station, TX, USA).

## Results

The analytic cohort comprised 428,207 participants after excluding individuals with prior sepsis (N = 961) and missing TyG data (N = 73,295)(**Supplementary Figure 1**). Over a median follow-up of 13.04 years (IQR: 12.43-13.85; 5,581,994 person-years), 12,410 sepsis cases (incidence rate: 2.22/1,000 person-years) and 6,291 sepsis-related mortalities (mortality rate: 0.93/1,000 person-years) were recorded. As shown in **Table 1**, compared to sepsis-free controls, both sepsis cases and sepsis-related mortality groups displayed progressively elevated TyG index (8.71 ± 0.57 vs. 8.86 ± 0.61 vs. 8.86 ± 0.61), older mean age (56.43 ± 8.09 vs. 60.58 ± 6.93 vs. 61.74 ± 6.26 years), higher White ethnicity proportion (88.26% vs. 89.41% vs. 90.05%), increased TDI scores, greater tobacco and alcohol consumption, lower women proportion (54.18% vs. 42.77% vs. 40.57%), reduced diet scores (2.12 ± 1.02 vs. 2.07 ± 1.01 vs. 2.06 ± 0.99), and elevated retirement rates. Cardiovascular risk stratification demonstrated graded increases in diabetes prevalence (4.94% vs. 13.72% vs. 14.51%), BMI (27.39 ± 4.75 vs. 28.95 ± 5.56 vs. 28.79 ± 5.58 kg/m²), SBP (139.70 ± 19.63 vs. 143.35 ± 20.17 vs. 144.27 ± 20.37 mmHg), and DBP (82.25 ± 10.68 vs. 82.55 ± 11.05 vs. 82.30 ± 11.15 mmHg); Conversely, HDL (1.45 ± 0.38 vs. 1.36 ± 0.39 vs. 1.35 ± 0.40 mmol/L) and LDL (3.56 ± 0.87 vs. 3.39 ± 0.92 vs. 3.35 ± 0.94 mmol/L) displayed decreasing trends across the groups.

**Table 1:** Baseline characteristics of included participants.

Variables	Without sepsis	Sepsis	Sepsis with mortality	P-value
N	415797	12410	6291	
Age (years)	56.43 ± 8.09	60.58 ± 6.93	61.74 ± 6.26	<0.001
Race, White (%)	366969 (88.26%)	11096 (89.41%)	5665 (90.05%)	<0.001
Sex, Women (%)	225294 (54.18%)	5308 (42.77%)	2552 (40.57%)	<0.001
Diabetes history(%)	20523 (4.94%)	1703 (13.72%)	913 (14.51%)	<0.001
TDI	-1.33 ± 3.08	-0.86 ± 3.28	-0.83 ± 3.31	<0.001
BMI (kg/m^2^)	27.39 ± 4.75	28.95 ± 5.56	28.79 ± 5.58	<0.001
MET	1041.84 ± 1087.02	982.73 ± 1062.32	972.83 ± 1054.68	<0.001
Diet score	2.12 ± 1.02	2.07 ± 1.01	2.06 ± 0.99	<0.001
Smoking history(%)				<0.001
Never	227750 (54.77%)	5231 (42.15%)	2458 (39.07%)	
Current	142700 (34.32%)	5210 (41.98%)	2728 (43.36%)	
Quit or no answer	45347 (10.91%)	1969 (15.87%)	1105 (17.57%)	
Education level (%)				<0.001
College or University	159234 (38.30%)	4764 (38.39%)	2338 (37.16%)	
Below	135448 (32.58%)	2914 (23.48%)	1424 (22.64%)	
None or no answer	121115 (29.13%)	4732 (38.13%)	2529 (40.20%)	
Employment status				<0.001
In employment	38781 (9.33%)	1566 (12.62%)	758 (12.05%)	
Retired	240473 (57.83%)	4651 (37.48%)	2036 (32.36%)	
Unemployed or no answer	136543 (32.84%)	6193 (49.90%)	3497 (55.59%)	
Alcohol consumption				<0.001
Current	18180 (4.37%)	674 (5.43%)	337 (5.36%)	
Previous	14628 (3.52%)	743 (5.99%)	397 (6.31%)	
No	382989 (92.11%)	10993 (88.58%)	5557 (88.33%)	
TyG	8.71 ± 0.57	8.86 ± 0.61	8.86 ± 0.61	<0.001
SBP (mmHg)	139.70 ± 19.63	143.35 ± 20.17	144.27 ± 20.37	<0.001
DBP (mmHg)	82.25 ± 10.68	82.55 ± 11.05	82.30 ± 11.15	0.692
HDL (mmol/L)	1.45 ± 0.38	1.36 ± 0.39	1.35 ± 0.40	<0.001
LDL (mmol/L)	3.56 ± 0.87	3.39 ± 0.92	3.35 ± 0.94	<0.001

Continuous data shown are mean ± SD; categorical data are shown as n (%). TyG, triglyceride-glucose index; HR, heart rate; TDI, Townsend indicator of deprivation; BMI, body mass index; DBP, diastolic blood pressure; SBP, systolic blood pressure; MET, metabolic equivalent of task; HDL, high density lipoprotein; LDL low density lipoprotein.

**Figure 1:**
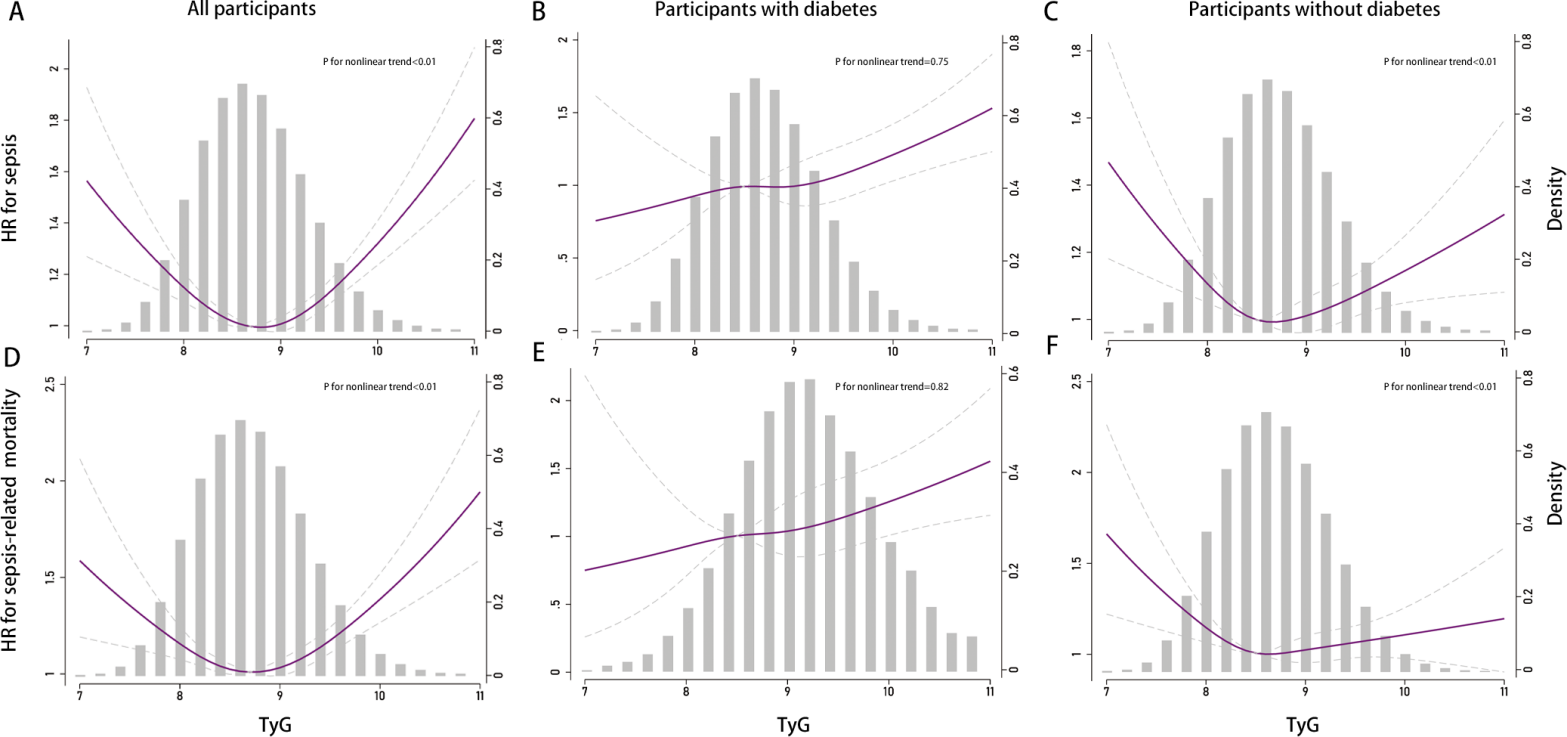
Dose-response associations of the TyG index with sepsis incidence and sepsis-related mortality. **(A)** TyG index and sepsis risk in all participants. **(B)** TyG index and sepsis risk in participants with diabetes. **(C)** TyG index and sepsis risk in participants without diabetes. **(D)** TyG index and sepsis-related mortality in all participants. **(E)** TyG index and sepsis-related mortality in participants with diabetes **(F)** TyG index and sepsis-related mortality in participants without diabetes. The purple solid line and gray dashed lines represent the hazard ratio (HR) and 95% CI, respectively. Gray histograms illustrate the distribution of the TyG index across the study population. All models were adjusted for covariates in Model 2: age, sex, race, TDI, MET, diet score, tobacco/alcohol consumption, education level, employment status, and BMI. Results are expressed as HRs with 95% CIs. TyG index, triglyceride-glucose index; TDI, Townsend Deprivation Index; MET, metabolic equivalent of task; BMI, body mass index; HR, hazard ratio; CI, confidence interval.

**Table 2** presents the associations between TyG index and sepsis and sepsis-related mortality incidence. In the overall population, unadjusted analysis revealed a positive dose-response relationship, with sepsis risk increasing proportionally to TyG index elevation (P for trend <0.001). However, this association was substantially attenuated after adjustment for Model 2 covariates. There was an elevation in risk per 1 - unit increment in the TyG index (HR: 1.12, 95% CI: 1.09–1.16, Model 2, **Table 2**). Restricted cubic spline analysis demonstrated a U-shaped association (P for nonlinear trend <0.05), with a TyG value of 8.7 identified as the inflection point of minimal sepsis risk (**Figure 1A**). Subgroup analyses revealed distinct patterns stratified by diabetes status. Among individuals with diabetes, the risk of sepsis increased in males with each higher quartile of the TyG index across all models. Compared with those in the first quartile of TyG index, individuals in the fourth quartile of TyG index had an HR of 1.37 (95% CI: 1.11–1.68, Model 2) for sepsis incidence. Restricted cubic spline analysis demonstrated a robust linear association (P for nonlinear trend = 0.75; **Figure 1B**), where each 1-unit increase in TyG index corresponded to an 18% elevated sepsis risk (HR: 1.18, 95% CI: 1.10–1.27, Model 2; **Table 2**). In contrast, among non-diabetic individuals, only the second TyG quartile (Q2) showed a hazard ratio slightly below 1 in Model 2, whereas Q3 and Q4 were not statistically significant (P > 0.05 for both), and the overall P for trend was also nonsignificant. Similarly, the continuous TyG analysis demonstrated no significant association with sepsis risk (adjusted HR: 1.02, 95% CI: 0.98–1.06).Nonlinear analysis in this subgroup confirmed a U-shaped pattern (P for nonlinear trend <0.05) mirroring the curve of the overall population (**Figure 1C**).

**Table 2:** Association between TyG and sepsis risk after adjusting for variables.

TyG	N	Incident rate*	Unadjusted		Model 1		Model 2	
			HR (95% CI)	P value	HR (95% CI)	P value	HR (95% CI)	P value
All								
Continues			1.54(1.50,1.59)	<0.01	1.34(1.30,1.38)	<0.01	1.12(1.09,1.16)	<0.01
Quantiles								
Q1	107,130	4.42	Ref		Ref		Ref	
Q2	107,061	5.43	1.23(1.16,1.30)	<0.01	1.03(0.98,1.09)	0.21	0.94(0.89,0.99)	<0.01
Q3	107,053	6.33	1.43(1.36,1.51)	<0.01	1.13(1.07,1.19)	<0.01	0.95(0.90,1.00)	0.02
Q4	106,963	8.20	1.86(1.76,1.95)	<0.01	1.42(1.35,1.50)	<0.01	1.08(1.03,1.14)	0.12
P for trend		<0.01		<0.01		<0.01		0.01
With diabetes								
Continues			1.27(1.19,1.35)	<0.01	1.27(1.19,1.36)	<0.01	1.18(1.10,1.27)	<0.01
Quantiles								
Q1	5,556	13.6	Ref		Ref		Ref	
Q2	5,557	15.0	1.11(0.96,1.28)	0.46	0.99(0.86,1.15)	0.91	1.03(0.87,1.22)	0.73
Q3	5,557	17.1	1.26(1.10,1.45)	<0.01	1.12(0.97,1.29)	0.12	1.05(0.89,1.25)	0.89
Q4	5,556	20.1	1.49(1.30,1.71)	<0.01	1.32(1.15,1.52)	<0.01	1.37(1.11,1.68)	<0.01
P for trend		<0.01		<0.01		<0.01		<0.01
Without diabetes								
Continues			1.38(1.34,1.43)	<0.01	1.19(1.15,1.23)	<0.01	1.02(0.98,1.06)	1.00
Quantiles								
Q1	101,495	4.27	Ref		Ref		Ref	
Q2	101,495	5.11	1.20(1.13,1.27)	<0.01	1.01(0.95,1.07)	0.82	0.93(0.88,0.98)	0.04
Q3	101,496	5.95	1.39(1.32,1.47)	<0.01	1.10(1.04,1.16)	<0.01	0.94(0.89,1.00)	0.36
Q4	101,495	6.83	1.60(1.52,1.69)	<0.01	1.24(1.17,1.31)	<0.01	0.99(0.94,1.05)	0.25
P for trend		<0.01		<0.01		<0.01		0.68

Cox proportional hazards models were used to assess the relationship between the TyG index – measured both as a 1-unit increment and in quantiles – and sepsis risk. Model 1 was adjusted for age, sex, and race; Model 2 additionally included the TDI, MET, diet score, tobacco/alcohol consumption, education level, employment status, and BMI. Results are expressed as HRs with 95% CIs.

TyG, triglyceride-glucose index; SD, standard deviation; TDI, Townsend Deprivation Index; MET, metabolic equivalent of task; BMI, body mass index; HR, hazard ratio; CI, confidence interval; Ref, reference.

• Incidence rate per 1,000,000 person-years.

The TyG index demonstrated comparable directional associations with both sepsis-related mortality and sepsis incidence, though population-level analyses revealed no significant association between TyG index and mortality. Restricted cubic spline analysis identified a U-shaped mortality pattern (**Figure 1D**), yet the overall association remained statistically non-significant (P>0.05) in both quartile-based comparisons and continuous analyses after adjusting for Model 2 covariates (**Table 3**). Subgroup stratification revealed similar diabetes-specific patterns: Diabetic individuals maintained a persistent linear dose-response relationship (**Figure 1E**). After full adjustment, each 1-unit elevation in the TyG index corresponded to an 18% increased mortality risk (HR: 1.16, 95% CI: 1.05 - 1.27, Model 2; **Table 3**),. In contrast, in non-diabetic populations, there was no significant association between the TyG index and mortality in either categorical or continuous analyses (**Table 3**). Nonlinear analysis in this subgroup revealed an asymmetric flattened U-curve (P for nonlinear trend < 0.01). The curve was characterized by a steep reduction in mortality risk until the TyG index reached 8.6, followed by a marginal elevation in risk beyond this threshold (**Figure 1F**).

**Table 3:** Association between TyG and sepsis-related mortality after adjusting for variables.

TyG	N	Incident rate*	Unadjusted		Model 1		Model 2	
			HR (95% CI)	P value	HR (95% CI)	P value	HR (95% CI)	P value
All								
Continues			1.55(1.49,1.62)	<0.01	1.31(1.25,1.37)	<0.01	1.10(1.05,1.16)	<0.01
Quantiles								
Q1	107,130	2.20	Ref		Ref		Ref	
Q2	107,061	2.74	1.24(1.15,1.34)	<0.01	1.00(0.93,1.09)	0.92	0.92(0.85,0.99)	0.03
Q3	107,053	3.19	1.45(1.34,1.56)	<0.01	1.08(1.00,1.16)	0.05	0.92(0.85,1.00)	0.05
Q4	106,963	4.16	1.89(1.76,2.03)	<0.01	1.36(1.26,1.46)	<0.01	1.05(0.97,1.13)	0.40
P for trend		<0.01		<0.01		<0.01		0.12
With diabetes								
Continues			1.27(1.16,1.38)	<0.01	1.26(1.15,1.38)	<0.01	1.16(1.05,1.27)	<0.01
Qertiles								
Q1	5,556	7.33	Ref		Ref		Ref	
Q2	5,557	7.29	0.99(0.81,1.21)	0.93	0.93(0.76,1.13)	0.45	0.91(0.72,1.15)	0.42
Q3	5,557	9.47	1.29(1.07,1.56)	<0.01	1.21(1.00,1.47)	0.05	1.13(0.90,1.42)	0.28
Q4	5,556	10.60	1.45(1.20,1.74)	<0.01	1.39(1.16,1.68)	<0.01	1.27(1.02,1.59)	0.03
P for trend		<0.01		<0.01		<0.01		0.01
Without diabetes								
Continues			1.37(1.31,1.44)	<0.01	1.14(1.09,1.20)	<0.01	0.99(0.94,1.05)	0.78
Quantiles								
Q1	101,495	2.12	Ref		Ref		Ref	
Q2	101,495	2.54	1.20(1.11,1.30)	<0.01	0.97(0.89,1.05)	0.42	0.90(0.82,0.97)	0.01
Q3	101,496	3.03	1.43(1.32,1.55)	<0.01	1.06(0.98,1.15)	0.13	0.94(0.86,1.01)	0.21
Q4	101,495	3.37	1.59(1.47,1.72)	<0.01	1.16(1.07,1.26)	<0.01	0.95(0.87,1.03)	0.12
P for trend		<0.01		<0.01		<0.01		0.41

Cox proportional hazards models were used to assess the relationship between the TyG index – measured both as a 1-unit increment and in quantiles – and sepsis-related mortality. Model 1 was adjusted for age, sex, and race; Model 2 additionally included the TDI, MET, diet score, tobacco/alcohol consumption, education level, employment status, and BMI. Results are expressed as HRs with 95% CIs.

TyG, triglyceride-glucose index; SD, standard deviation; TDI, Townsend Deprivation Index; MET, metabolic equivalent of task; BMI, body mass index; HR, hazard ratio; CI, confidence interval; Ref, reference.

• Incidence rate per 1,000,000 person-years.

**Figure 2:**
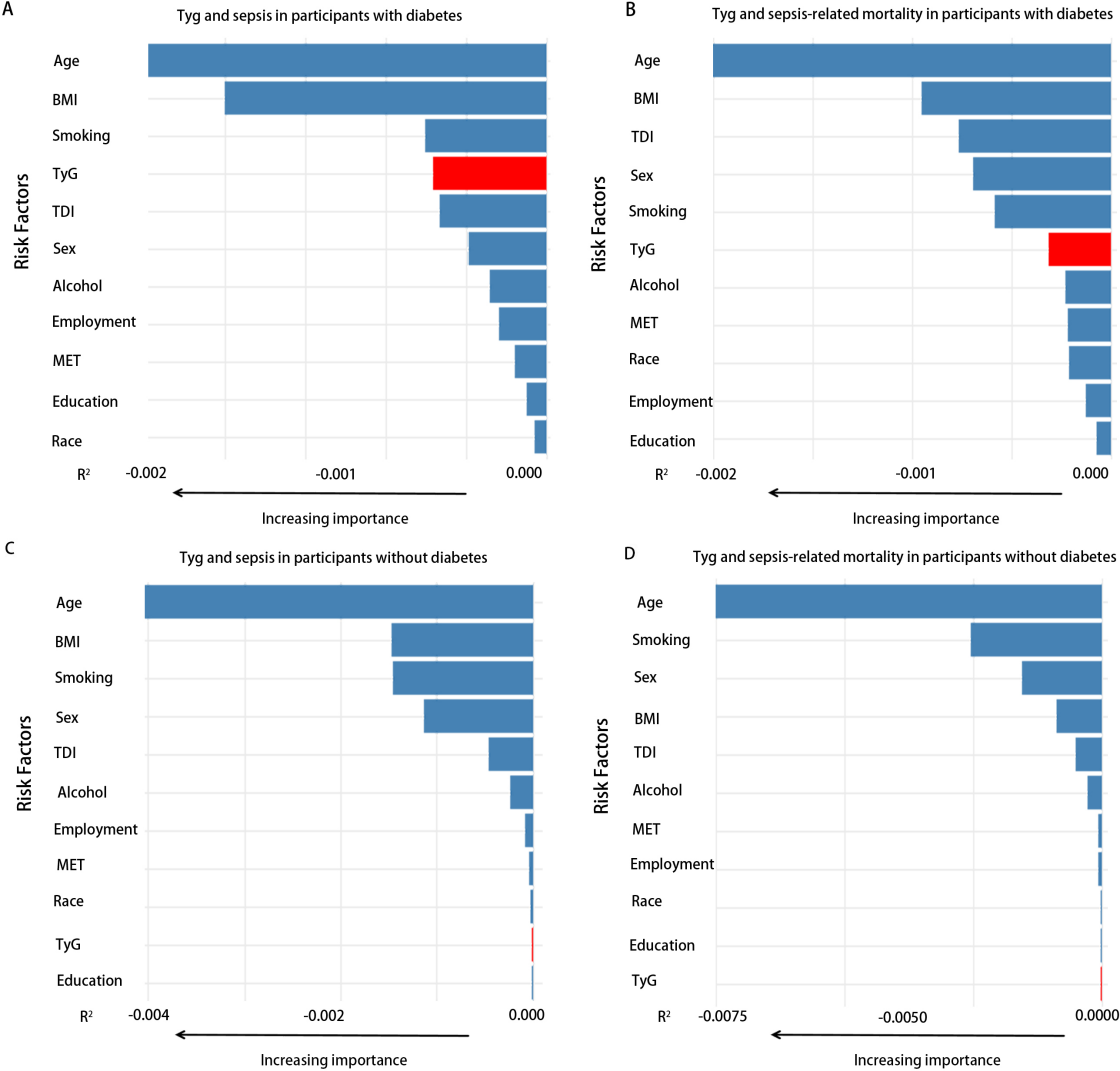
Relative predictive importance of the TyG index for sepsis outcomes. **(A)** Relative importance of the TyG index as the fourth most significant predictor of sepsis risk in Model 2. **(B)** Relative importance of the TyG index as the sixth most significant predictor of sepsis incidence in Model 2. **(C)** Relative importance of the TyG index as the second-least significant predictor of sepsis-related morbidity in Model 2. **(D)** Relative importance of the TyG index as the least most significant predictor of sepsis-related mortality in Model 2. The relative contributions of the TyG index were evaluated by comparing R² values in Model 2, which included the TyG index and covariates simultaneously. All models were adjusted for the same covariates as in Model 2: age, sex, race, TDI, MET, diet score, tobacco/alcohol consumption, education level, employment status, and BMI. Abbreviations: Same as in **Figure 1**.

To further evaluate the predictive value of the TyG index for sepsis and sepsis-related mortality, we compared the TyG index with covariates in Model 2 stratified by diabetes status. The predictive value of the TyG index differed sharply based on diabetes status. The TyG index ranked as the fourth most important predictor of sepsis risk (**Figure 2A**) and the sixth most important predictor for sepsis-related mortality (**Figure 2B**). However, in non-diabetic populations, the TyG index was the second-least important predictor of sepsis risk (**Figure 2C**) and the least important predictor for sepsis-related mortality (**Figure 2D**). To further validate the incremental predictive utility of the TyG index, we evaluated the model performance using Harrell’s C-index, the NRI, and the IDI. In line with our primary findings, the impact of the TyG index on predictive performance differed markedly according to diabetes status (**Table 4**; **Supplementary Table 1**). Among individuals with diabetes, the integration of the TyG index significantly enhanced all predictive metrics for both sepsis risk and sepsis-related mortality (**Table 4**; **Supplementary Table 1**). Specifically, for sepsis risk in the diabetes subgroup, the C-statistic rose from 0.6735 to 0.6761 (P < 0.01) when comparing the fully adjusted Model 2 with the TyG-integrated model (**Table 4**). This improvement was further supported by a significant NRI of 0.1091 (95% CI: 0.0647-0.1563, P < 0.01) and an IDI of 0.0156 (95% CI: 0.0065-0.0286, P < 0.01), indicating substantial enhancement in risk reclassification and discrimination. Similarly, for sepsis-related mortality in this group, the C-statistic increased from 0.6711 to 0.6737 (P < 0.01) (**Table 4**), with a corresponding NRI of 0.0924 (P < 0.01) and IDI of 0.0124 (P < 0.01). Conversely, among individuals without diabetes, the addition of the TyG index did not yield significant improvements in the C-statistic, NRI, or IDI for predicting either outcome in the fully adjusted model (all P > 0.05; **Supplementary Table 1**). These results reinforce that the TyG index provides meaningful incremental predictive value specifically for the diabetic population. To further explore the associations between the TyG index and the risks of sepsis and sepsis - related mortality among individuals with or without diabetes, we conducted stratified analyses by age (<60 years vs. ≥60 years), sex, and BMI categories (<30 kg/m² vs. ≥30 kg/m²). The findings demonstrated that the links between TyG index and the outcomes remained stable across all subgroups, with no evidence of significant effect modification by age, sex, or BMI categories, as reflected by interaction P-values exceeding 0.05 (**Supplementary Tables 2**, **3**). The robustness of these results was further validated through sensitivity analyses employing a competing risk model (**Supplementary Tables 4**, **5**). Additional sensitivity analyses—including the exclusion of sepsis cases during the first two years of follow-up, right-censoring participants at age 70, excluding those with incomplete data, and further adjusting for antihyperglycemic medications, LDL, HDL, SBP, CRP, DBP, and a history of CKD, and using the average TyG index from repeated measurements (**Supplementary Tables 6**, **7**)—provided further support for the robustness of the findings.

**Table 4:** Incremental predictive value of the TyG index.

Outcomes	Diabetes history	Harrell’s C-index	
		Estimate (95% CI)	P value
Sepsis	All		
	Model 1	0.6627(0.6561-0.6694)	Ref
	Model 1+TyG	0.6678(0.6613-0.6745)	<0.01
	Model 2	0.6921(0.6855-0.6986)	Ref
	Model 2+TyG	0.6927(0.6862-0.6993)	0.02
	With diabetes		
	Model 1	0.6317(0.6108-0.6526)	Ref
	Model 1+TyG	0.6389(0.6186-0.6592)	<0.01
	Model 2	0.6735(0.6534-0.6937)	Ref
	Model 2+TyG	0.6761(0.6561-0.6961)	<0.01
	Without diabetes		
	Model 1	0.6579(0.6506-0.6651)	Ref
	Model 1+TyG	0.6597(0.6524-0.6669)	<0.01
	Model 2	0.6825(0.6753-0.6896)	Ref
	Model 2+TyG	0.6825(0.6753-0.6897)	0.22
Sepsis-related mortality	All		
	Model 1	0.7039(0.7038-0.7209)	Ref
	Model 1+TyG	0.7124(0.7039-0.7210)	<0.01
	Model 2	0.7336(0.7252-0.7420)	Ref
	Model 2+TyG	0.7340(0.7256-0.7424)	0.16
	With diabetes		
	Model 1	0.6319(0.6108-0.6530)	Ref
	Model 1+TyG	0.6388(0.6183-0.6593)	0.05
	Model 2	0.6711(0.6508-0.6916)	Ref
	Model 2+TyG	0.6737(0.6534-0.6940)	<0.01
	Without diabetes		
	Model 1	0.7062(0.6968-0.7156)	Ref
	Model 1+TyG	0.7067(0.6974-0.7161)	0.30
	Model 2	0.7273(0.7180-0.7365)	Ref
	Model 2+TyG	0.7273(0.7180-0.7365)	0.69

Cox proportional hazards models were used to assess the relationship between the TyG index – measured both as a 1-unit increment– and sepsis-related mortality. Harrell’s C-index was employed to compare the predictive performance of models with and without the TyG index. Model 1 was adjusted for age, sex, and race; Model 2 additionally included the TDI, MET, diet score, tobacco/alcohol consumption, education level, employment status, and BMI. Results are expressed as HRs with 95% CIs.

TyG, triglyceride-glucose index; SD, standard deviation; TDI, Townsend Deprivation Index; MET, metabolic equivalent of task; BMI, body mass index; HR, hazard ratio; CI, confidence interval; Ref, reference.

## Discussion

To the best of our knowledge, this study is the first prospective cohort to date investigating the association between the TyG index and sepsis as well as sepsis-related mortality in the general population. We observed several notable findings: 1) The association between the TyG index and sepsis/sepsis-related mortality was significantly modified by diabetes status; 2) In diabetic participants, a linear positive association was observed, where higher TyG indices were consistently linked to increased risks of sepsis and sepsis-related mortality. Furthermore, incorporating the TyG index improved the predictive performance for these outcomes; 3) In contrast, a U-shaped relationship was observed in non-diabetic individuals, with both low and high TyG indices associated with higher mortality risk. Notably, the TyG index did not improve the prediction of sepsis incidence or sepsis-related mortality.

The relationship between TyG index and sepsis incidence in the general population remains poorly characterized, as most prior investigations have predominantly focused on critically ill cohorts and established sepsis patients, yielding inconsistent conclusions (4–7, 15). Our study addresses this knowledge gap by elucidating the diabetes-dependent modulation of TyG-sepsis associations in population-based analyses. Notably, diabetic individuals exhibited distinct metabolic profiles characterized by obesity-related chronic inflammation (16), dyslipidemia (17), and sustained hyperglycemia (18) – features intrinsically linked to both elevated TyG values and sepsis susceptibility (19–21). In contrast, non-diabetic sepsis patients demonstrated a biphasic TyG-outcome relationship reflecting the dynamic equilibrium between acute metabolic stress and subclinical insulin resistance. Low TyG values may indicate maladaptive hypometabolism and malnutrition-related immunosuppression, impairing leukocyte glucose uptake, cytokine production, and compromise innate immune responses (22, 23). While our findings highlight the association between the TyG index and sepsis risk, the underlying biological mechanisms remain to be fully elucidated. It is theoretically possible that low TyG levels may reflect pre-existing sarcopenia or frailty, which are known to exacerbate sepsis severity. Conversely, high TyG levels might reflect lipid-driven inflammation and metabolic dysregulation, potentially involving pathways such as ceramide accumulation or mitochondrial dysfunction (24). However, as these specific biomarkers and metabolic flux were not directly measured in the present study, these mechanistic interpretations remain hypothetical. This dual-risk pattern suggests a complex interplay between energy metabolism and immune competence, but further studies incorporating direct nutritional assessments and mediation analyses are essential to confirm these pathways (5).

Our study further strengthens the epidemiological evidence by dissociating the baseline TyG index from the acute metabolic perturbations inherent to sepsis, thereby providing a clearer temporal framework to address causality. By focusing on pre-sepsis TyG values, we circumvent the bidirectional confounding where sepsis-induced insulin resistance could transiently elevate TyG levels, potentially misattributing prognostic significance to a marker influenced by the disease itself. This design also mitigates the confounding effects of therapeutic interventions during sepsis (e.g., insulin therapy, parenteral nutrition), which can acutely alter glucose and lipid metabolism and obscure the intrinsic association between baseline metabolic status and sepsis risk.

From a public health perspective, our findings underscore the clinical utility of integrating the TyG index into routine metabolic assessments for sepsis risk stratification, particularly individuals with diabetes. Elevated baseline TyG levels in individuals with diabetes—a proxy for insulin resistance, dyslipidemia, and chronic inflammation—serve as a modifiable biomarker to identify those requiring targeted interventions to mitigate sepsis susceptibility (3, 25). Lifestyle modifications (e.g., dietary counseling, exercise) or pharmacological strategies (e.g., metformin, GLP-1 agonists) aimed at improving insulin sensitivity and reducing pro-inflammatory lipid metabolites (e.g., ceramides) could potentially disrupt the metabolic pathways linking TyG to sepsis in this high-risk group (26, 27). In non-diabetic cohorts, the U-shaped TyG-sepsis mortality association highlights the need for nuanced interventions: individuals with low TyG levels may benefit from nutritional optimization to address hypometabolic states and restore leukocyte glucose utilization, while those with elevated TyG levels require strategies to alleviate insulin resistance and lipid toxicity. This dual-pronged approach emphasizes the importance of diabetes-specific risk stratification, as metabolic derangements in diabetic versus non-diabetic populations exhibit distinct mechanistic links to sepsis pathogenesis.

### Limitation

Our study has several limitations. First, as an observational study based on the UK Biobank, the potential for residual confounding and the inability to infer direct causality remain inherent limitations; however, the robustness of our findings across multiple stringent sensitivity analyses, alongside the large-scale and high-quality nature of this cohort, provides strong internal validity for the observed associations. Second, our exclusively UK-based sample limits generalizability to other ethnic groups with distinct lifestyles and sepsis risk profiles. Third, as the UK Biobank participants tend to be healthier than the general population (‘healthy volunteer’ effect), caution is warranted when generalizing these findings to broader or more diverse populations. Fourth, We acknowledge that the absolute increase in the C-statistic is modest. However, it is noteworthy that the TyG index significantly improved the model’s predictive performance even after rigorous adjustment for a comprehensive set of established clinical risk factors and potential confounders. This suggests that the TyG index captures unique metabolic information not fully accounted for by traditional variables. While the marginal increment may seem small, such gains are common in large-scale epidemiological studies where baseline models are already strong. Finally, the TyG index was calculated based on a single baseline measurement, which may not reflect longitudinal changes in metabolic status during the follow-up period; however, baseline metabolic markers in the UK Biobank have been widely utilized and validated as reliable indicators of long-term health outcomes in large-scale prospective studies.

## Conclusion

To our knowledge, this prospective cohort study is among the first to investigate the association between the TyG index and sepsis, as well as sepsis-related mortality, in the general population. Our findings highlight a diabetes-status-dependent relationship between the TyG index and sepsis outcomes. In individuals with diabetes, higher TyG indices were linearly and positively associated with increased risks of both sepsis and sepsis-related mortality, and incorporating the TyG index improved predictive models for these outcomes. This suggests that the TyG index may serve as a valuable risk-stratification tool in diabetic populations, warranting further validation in independent cohorts.

## Data Availability

The original contributions presented in the study are included in the article/**Supplementary Material**. Further inquiries can be directed to the corresponding author.
