# Supplementary material for: Association of TyG index with sepsis incidence and mortality: a prospective study with diabetes stratification
Source: Front Endocrinol (Lausanne). 2026 May 28;17:1834832. doi: 10.3389/fendo.2026.1834832 (PMC13253386; doi:10.3389/fendo.2026.1834832)
Supplement: Supplementary file 1 [file Table1.docx]

**Tables**

Supplementary Table 1. Incremental predictive value of the TyG index evaluated by ∆NRI and ∆IDI

Supplementary Table 2. Subgroup analysis of the association between TyG index and sepsis risk.

Supplementary Table 3. Subgroup analysis of the association between TyG index and sepsis-related mortality.

Supplementary Table 4. Association between TyG and sepsis risk using the competing risk model.

Supplementary Table 5. Association between TyG and sepsis-related mortality risk using the competing risk model.

Supplementary Table 6. Sensitivity analyses of associations between TyG index with sepsis using different exclusion criteria and adjusted models.

Supplementary Table 7. Sensitivity analyses of associations between TyG index with sepsis-related mortality using different exclusion criteria and adjusted models.

Figures

Supplementary Figure 1. The flow chat of present study

**Supplementary Table 1. Incremental predictive value of the TyG index evaluated by ∆NRI and ∆IDI**

| **Outcomes** | **Diabetes history** | **∆NRI** | | **∆IDI** | |
| --- | --- | --- | --- | --- | --- |
|  |  | **Estimate (95% CI)** | **P value** | **Estimate (95% CI)** | **P value** |
| **Sepsis** | **All** |  | |  |  |
|  | Model 1 | Ref | Ref |  |  |
|  | Model 1+TyG | 0.1448 (0.1266-0.1609) | <0.01 | 0.0305 (0.0249-0.0359) | <0.01 |
|  | Model 2 | Ref | Ref |  |  |
|  | Model 2+TyG | 0.0787 (0.0625-0.0933) | 0.02 | 0.0056 (0.0037-0.0089) | <0.01 |
|  | **With diabetes** |  | |  |  |
|  | Model 1 | Ref | Ref |  |  |
|  | Model 1+TyG | 0.1406 (0.0909-0.1859) | <0.01 | 0.0056 (0.0037-0.0089) | <0.01 |
|  | Model 2 | Ref | Ref |  |  |
|  | Model 2+TyG | 0.1091 (0.0647-0.1563) | <0.01 | 0.0156 (0.0065-0.0286**)** | <0.01 |
|  | **Without diabetes** |  | |  |  |
|  | Model 1 | Ref | Ref |  |  |
|  | Model 1+TyG | 0.0961 (0.0762-0.1155) | <0.01 | 0.0102 (0.0072-0.0142) | <0.01 |
|  | Model 2 | Ref | Ref |  |  |
|  | Model 2+TyG | 0.0444 (-0.0419-0.0647) | 0.50 | 0.0003 (-0.0002-0.0015) | 0.50 |
| **Sepsis-related mortality** | **All** |  |  |  |  |
|  | Model 1 | Ref | Ref |  |  |
|  | Model 1+TyG | 0.1379 (0.1134-0.1593) | <0.01 | 0.0275 (0.0206-0.0367) | <0.01 |
|  | Model 2 | Ref | Ref |  |  |
|  | Model 2+TyG | 0.0822 (0.0605-0.1028) | <0.01 | 0.0048 (0.0023-0.0087) | <0.01 |
|  | **With diabetes** |  |  |  |  |
|  | Model 1 | Ref | Ref |  |  |
|  | Model 1+TyG | 0.1398 (0.0755-0.2099) | <0.01 | 0.0292 (0.0123-0.0503) | <0.01 |
|  | Model 2 | Ref | Ref |  |  |
|  | Model 2+TyG | 0.0924 (0.0296-0.1577) | <0.01 | 0.0124 (0.0019-0.0287) | <0.01 |
|  | **Without diabetes** |  |  |  |  |
|  | Model 1 | Ref | Ref |  |  |
|  | Model 1+TyG | 0.0876 (0.0678-0.1125) | <0.01 | 0.0072 (0.0035-0.0115) | <0.01 |
|  | Model 2 | Ref | Ref |  |  |
|  | Model 2+TyG | -0.0426 (-0.0529-0.0646) | 0.78 | -0.0001 (-0.0004-0.0014) | 0.78 |

*Note: Cox proportional hazards models were used to assess the relationship between the TyG index – measured both as a 1-unit increment– and sepsis-related mortality. ∆NRI and ∆IDI was employed to compare the predictive performance of models with and without the TyG index. Model 1 was adjusted for age, sex, and race; Model 2 additionally included the TDI, MET, diet score, tobacco/alcohol consumption, education level, employment status, and BMI. Results are expressed as HRs with 95% CIs.*

*Abbreviations: TyG, triglyceride-glucose index; SD, standard deviation; TDI, Townsend Deprivation Index; MET, metabolic equivalent of task; BMI, body mass index; HR, hazard ratio; CI, confidence interval; Ref, reference.*

**Supplementary Table 2. Subgroup analysis of the association between TyG index and sepsis risk.**

|  | **Subgroups** | | **HR (95% CI)** | **P for interaction** |
| --- | --- | --- | --- | --- |
| **All** | Age | ≤60 | 1.11(1.05,1.18) | 0.43 |
|  |  | >60 | 1.11(1.06,1.17) |  |
|  | Sex | Women | 1.21(1.14,1.29) | 0.11 |
|  |  | Men | 1.07(1.02,1.12) |  |
|  | BMI groups | ≤30 | 1.08(1.03,1.13) | <0.01* |
|  |  | >30 | 1.18(1.11,1.26) |  |
| **With diabetes** | Age | ≤60 | 1.19(1.04,1.35) | 0.77 |
|  |  | >60 | 1.17(1.05,1.30) |  |
|  | Sex | Women | 1.26(1.08,1.47) | 0.19 |
|  |  | Men | 1.15(1.04,1.26) |  |
|  | BMI groups | ≤30 | 1.13(1.01,1.28) | 0.12 |
|  |  | >30 | 1.24(1.12,1.38) |  |
| **Without diabetes** | Age | ≤60 | 1.01(0.95,1.08) | 0.33 |
|  |  | >60 | 1.01(0.96,1.07) |  |
|  | Sex | Women | 1.11(1.04,1.19) | 0.07 |
|  |  | Men | 0.96(0.91,1.02) |  |
|  | BMI groups | ≤30 | 1.02(0.97,1.07) | 0.92 |
|  |  | >30 | 1.00(0.92,1.08) |  |

*Note: Cox proportional hazards models were used to assess the relationship between the TyG index – measured both as a 1-unit increment – and sepsis risk. Model 2 was adjusted for age, sex, race TDI, MET, diet score, tobacco/alcohol consumption, education level, employment status, and BMI. Results are expressed as HRs with 95% CIs.*

*Abbreviations: TyG, triglyceride-glucose index; SD, standard deviation; TDI, Townsend Deprivation Index; MET, metabolic equivalent of task; BMI, body mass index; HR, hazard ratio; CI, confidence interval.*

**Supplementary Table 3. Subgroup analysis of the association between TyG index and sepsis-related mortality.**

|  | **Subgroups** | | **HR (95% CI)** | **P for interaction** | |
| --- | --- | --- | --- | --- | --- |
| **All** | Age | ≤60 | 1.07(0.98,1.17) | | 0.49 |
|  |  | >60 | 1.11(1.03,1.18) | |  |
|  | Sex | Women | 1.19(1.09,1.30) | | 0.30 |
|  |  | Men | 1.06(0.99,1.13) | |  |
|  | BMI groups | ≤30 | 1.09(1.02,1.17) | | 0.03* |
|  |  | >30 | 1.16(1.06,1.27) | |  |
| **With diabetes** | Age | ≤60 | 1.20(0.99,1.44) | | 0.77 |
|  |  | >60 | 1.18(1.03,1.35) | |  |
|  | Sex | Women | 1.33(1.07,1.66) | | 0.20 |
|  |  | Men | 1.14(1.00,1.30) | |  |
|  | BMI groups | ≤30 | 1.20(1.01,1.43) | | 0.42 |
|  |  | >30 | 1.21(1.05,1.40) | |  |
| **Without diabetes** | Age | ≤60 | 0.95(0.85,1.05) | | 0.22 |
|  |  | >60 | 1.00(0.92,1.08) | |  |
|  | Sex | Women | 1.08(0.98,1.19) | | 0.14 |
|  |  | Men | 0.94(0.87,1.01) | |  |
|  | BMI groups | ≤30 | 1.01(0.94,1.09) | | 0.59 |
|  |  | >30 | 0.95(0.84,1.06) | |  |

*Note: Cox proportional hazards models were used to assess the relationship between the TyG index – measured both as a 1-unit increment – and sepsis-related mortality. Model 2 was adjusted for age, sex, race TDI, MET, diet score, tobacco/alcohol consumption, education level, employment status, and BMI. Results are expressed as HRs with 95% CIs.*

*Abbreviations: TyG, triglyceride-glucose index; SD, standard deviation; TDI, Townsend Deprivation Index; MET, metabolic equivalent of task; BMI, body mass index; HR, hazard ratio; CI, confidence interval.*

** p<0.05.*

**Supplementary Table 4. Association between TyG and sepsis risk using the competing risk model.**

| **TyG index** | **N** | **Incident rate*** | **HR (95% CI)** | |
| --- | --- | --- | --- | --- |
|  |  |  | **Model 1** | **Mdel 2** |
| **All** |  |  |  |  |
| Continues |  |  | 1.34(1.30,1.38) | 1.12(1.09,1.17) |
| Quantiles |  |  |  |  |
| Q1 | 107,130 | 4.42 | Ref | Ref |
| Q2 | 107,061 | 5.43 | 1.03(0.98,1.09) | 0.94(0.89,0.99) |
| Q3 | 107,053 | 6.33 | 1.12(1.06,1.19) | 0.95(0.90,1.01) |
| Q4 | 106,963 | 8.20 | 1.41(1.34,1.49) | 1.09(1.03,1.15) |
| P for trend |  | <0.01 | <0.01 | <0.01 |
| **With diabetes** |  |  |  |  |
| Continues |  |  | 1.19(1.14,1.23) | 1.01(0.98,1.06) |
| Quantiles |  |  |  |  |
| Q1 | 5,556 | 13.6 | Ref | Ref |
| Q2 | 5,557 | 15.0 | 1.01(0.95,1.07) | 0.94(0.89,1.00) |
| Q3 | 5,557 | 17.1 | 1.09(1.03,1.15) | 0.95(0.90,1.01) |
| Q4 | 5,556 | 20.1 | 1.23(1.17,1.31) | 1.09(1.03,1.15) |
| P for trend |  | <0.01 | <0.01 | 0.78 |
| **Without diabetes** |  |  |  |  |
| Continues |  |  | 1.15(1.09,1.21) | 1.18(1.10,1.27) |
| Quantiles |  |  |  |  |
| Q1 | 101,495 | 4.27 | Ref | Ref |
| Q2 | 101,495 | 5.11 | 1.35(1.06,1.70) | 1.25(0.98,1.58) |
| Q3 | 101,496 | 5.95 | 1.21(0.97,1.51) | 1.09(0.87,1.37) |
| Q4 | 101,495 | 6.83 | 1.59(1.30,1.95) | 1.37(1.11,1.68) |
| P for trend |  | <0.01 | <0.01 | <0.01 |

*Note: Competing risk model were used to assess the relationship between the TyG index – measured both as a 1-unit increment and in quantiles – and sepsis risk. Model 1 was adjusted for age, sex, and race; Model 2 additionally included the TDI, MET, diet score, tobacco/alcohol consumption, education level, employment status, and BMI. Results are expressed as HRs with 95% CIs.*

*Abbreviations: TyG, triglyceride-glucose index; SD, standard deviation; TDI, Townsend Deprivation Index; MET, metabolic equivalent of task; BMI, body mass index; HR, hazard ratio; CI, confidence interval; Ref, reference.*

*• Incidence rate per 1,000,000 person-years.*

**Supplementary Table 5. Association between TyG and sepsis-related mortality risk using the competing risk model.**

| **TyG index** | **N** | **Incident rate*** | **HR (95% CI)** | |
| --- | --- | --- | --- | --- |
|  |  |  | **Model 1** | **Model 2** |
| **All** |  |  |  |  |
| Continues |  |  | 1.31(1.25,1.37) | 1.07(0.98,1.12) |
| Quantiles |  |  |  |  |
| Q1 | 107,130 | 2.20 | Ref | Ref |
| Q2 | 107,061 | 2.74 | 1.00(0.93,1.08) | 0.92(0.85,1.00) |
| Q3 | 107,053 | 3.19 | 1.07(0.99,1.16) | 0.93(0.86,1.00) |
| Q4 | 106,963 | 4.16 | 1.35(1.26,1.46) | 1.06(0.98,1.15) |
| P for trend |  | <0.01 | <0.01 | 0.06 |
| **With diabetes** |  |  |  |  |
| Continues |  |  | 1.14(1.06,1.23) | 1.09(1.01,1.17) |
| Quantiles |  |  |  |  |
| Q1 | 5,556 | 7.33 | Ref | Ref |
| Q2 | 5,557 | 7.29 | 1.54(1.12,2.13) | 1.40(1.02,1.94) |
| Q3 | 5,557 | 9.47 | 1.02(0.74,1.39) | 0.90(0.66,1.24) |
| Q4 | 5,556 | 10.60 | 1.64(1.23,2.17) | 1.38(1.04,1.83) |
| P for trend |  | <0.01 | <0.01 | <0.01 |
| **Without diabetes** |  |  |  |  |
| Continues |  |  | 1.05(1.03,1.09) | 0.99(0.94,1.05) |
| Quantiles |  |  |  |  |
| Q1 | 101,495 | 2.12 | Ref | Ref |
| Q2 | 101,495 | 2.54 | 0.96(0.89,1.05) | 0.90(0.82,0.97) |
| Q3 | 101,496 | 3.03 | 1.06(0.98,1.14) | 0.94(0.86,1.01) |
| Q4 | 101,495 | 3.37 | 1.16(1.07,1.25) | 0.95(0.87,1.03 |
| P for trend |  | <0.01 | <0.01 | 0.58 |

*Note: Competing risk model were used to assess the relationship between the TyG index – measured both as a 1-unit increment and in quantiles – and sepsis-related risk. Model 1 was adjusted for age, sex, and race; Model 2 additionally included the TDI, MET, diet score, tobacco/alcohol consumption, education level, employment status, and BMI. Results are expressed as HRs with 95% CIs.*

*Abbreviations: TyG, triglyceride-glucose index; SD, standard deviation; TDI, Townsend Deprivation Index; MET, metabolic equivalent of task; BMI, body mass index; HR, hazard ratio; CI, confidence interval; Ref, reference.*

*• Incidence rate per 1,000,000 person-years.*

**Supplementary Table 5. Sensitivity analyses of associations between TyG index with sepsis using different exclusion criteria and adjusted models.**

| **TyG index** | **Excluding**  **Sepsis < 2 years** | **Excluding age >70** | | **Complete-case analysis** | **Model 2+ antihyperglycemic agents+other covariates** | **Average TyG during follow-up period** |
| --- | --- | --- | --- | --- | --- | --- |
| **All** |  | |  |  |  |  |
| Continues | 1.12(1.09,1.16) | 1.12(1.09,1.16) | | 1.10(1.06,1.15) | 1.17(1.13,1.21) | 1.13 (1.09, 1.17) |
| Quantiles |  |  | |  |  |  |
| Q1 | Ref | Ref | | Ref | Ref | Ref |
| Q2 | 0.94(0.89,1.00) | 0.94(0.89,0.99) | | 0.91(0.85,0.97) | 0.97(0.93,1.04) | 0.94 (0.89, 1.00) |
| Q3 | 0.95(0.90,1.01) | 0.95(0.90,1.00) | | 0.93(0.87,0.99) | 1.01(0.96,1.07) | 0.96 (0.91, 1.02) |
| Q4 | 1.09(1.03,1.15) | 1.08(1.03,1.14) | | 1.05(0.99,1.12) | 1.17(1.09,1.23) | 1.10 (1.05, 1.16) |
| P for trend | <0.01 | <0.01 | | <0.01 | <0.01 | <0.01 |
| **With diabetes** |  | |  |  |  |  |
| Continues | 1.20(1.12,1.29) | 1.18(1.10,1.27) | | 1.18(1.09,1.27) | 1.21(1.12,1.31) | 1.19 (1.11, 1.29) |
| Quantiles |  |  | |  |  |  |
| Q1 | Ref | Ref | | Ref | Ref | Ref |
| Q2 | 1.34(1.05,1.73) | 1.25(0.98,1.58) | | 1.15(0.88,1.52) | 1.29(1.01,1.63) | 1.26 (0.98, 1.62) |
| Q3 | 1.18(0.93,1.50) | 1.09(0.87,1.37) | | 1.11(0.86,1.44) | 1.13(0.89,1.42) | 1.13 (0.89, 1.43) |
| Q4 | 1.47(1.18,1.83) | 1.37(1.11,1.68) | | 1.31(1.04,1.66) | 1.43(1.15,1.78) | 1.40 (1.12, 1.74) |
| P for trend | <0.01 | <0.01 | | <0.01 | <0.01 | <0.01 |
| **Without diabetes** |  | |  |  |  |  |
| Continues | 1.02(0.98,1.06) | 1.02(0.98,1.06) | | 0.99(0.96,1.04) | 1.05(0.99,1.10) | 1.02 (0.98, 1.07) |
| Quantiles |  |  | |  |  |  |
| Q1 | Ref | Ref | | Ref | Ref | Ref |
| Q2 | 0.92(0.87,0.98) | 0.93(0.88,0.98) | | 0.91(0.85,0.97) | 0.95(0.90,1.01) | 0.93 (0.88, 0.99) |
| Q3 | 0.94(0.89,1.00) | 0.94(0.89,1.00) | | 0.93(0.87,0.99) | 0.98(0.92,1.04) | 0.95 (0.89, 1.01) |
| Q4 | 0.99(0.93,1.05) | 0.99(0.93,1.05) | | 1.05(0.99,1.12) | 1.01(0.97,1.11) | 1.01 (0.96, 1.08) |
| P for trend | 0.82 | 0.79 | | 0.67 | 0.15 | 0.61 |

*Note: Cox proportional hazards models were used to assess the relationship between the TyG index – measured both as a 1-unit increment and in quantiles – and sepsis risk. Model 1 was adjusted for age, sex, and race; Model 2 additionally included the TDI, MET, diet score, tobacco/alcohol consumption, education level, employment status, and BMI. Other covariates included DBP, SBP, LDL, CRP, HDL, and CKD history.*

*Results are expressed as HRs with 95% CIs.*

*Abbreviations: TyG, triglyceride-glucose index; SD, standard deviation; TDI, Townsend Deprivation Index; MET, metabolic equivalent of task; BMI, body mass index; HR, hazard ratio; CI, confidence interval;DBP, diastolic blood pressure; SBP, systolic blood pressure; LDL low density lipoprotein; HDL, high density lipoprotein. Ref, reference.*

**Supplementary Table 6. Sensitivity analyses of associations between TyG index with sepsis-related mortality using different exclusion criteria and adjusted models.**

| **TyG** | **Excluding**  **Sepsis < 2 years** | | **Excluding age >70** | **Complete-case analysis** | **Model 2+ antihyperglycemic agents** | **Average TyG during follow-up period** |
| --- | --- | --- | --- | --- | --- | --- |
| **All** |  |  |  |  |  |  |
| Continues | 1.10(1.05,1.16) | | 1.10(1.05,1.16) | 1.02(0.99,1.05) | 1.14(1.10,1.22) | 1.09(1.05, 1.15) |
| Quantiles |  | |  |  |  |  |
| Q1 | Ref | | Ref | Ref | Ref | Ref |
| Q2 | 0.93(0.86,1.01) | | 0.92(0.85,0.99) | 0.90(0.83,0.99) | 0.97(0.89,1.05) | 0.93(0.86, 1.01) |
| Q3 | 0.93(0.86,1.01) | | 0.92(0.85,1.00) | 0.91(0.83,1.00) | 1.00(0.92,1.08) | 0.95(0.88, 1.02) |
| Q4 | 1.06(0.98,1.14) | | 1.05(0.97,1.13) | 1.04(0.95,1.13) | 1.15(1.04,1.24) | 1.08(0.99, 1.16) |
| P for trend | 0.04 | | 0.04 | 0.12 | <0.01 | 0.04 |
| **With diabetes** |  |  |  |  |  |  |
| Continues | 1.18(1.07,1.30) | | 1.16(1.05,1.27) | 1.12(1.02,1.22) | 1.18(1.07,1.32) | 1.16(1.05, 1.28) |
| Quantiles |  | |  |  |  |  |
| Q1 | Ref | | Ref | Ref | Ref | Ref |
| Q2 | 1.50(1.07,2.10) | | 1.40(1.02,1.94) | 1.37(0.94,2.01) | 1.48(1.07,2.05) | 1.44(1.03, 2.03) |
| Q3 | 0.96(0.69,1.33) | | 0.90(0.66,1.24) | 0.96(0.66,1.39) | 0.96(0.69,1.33) | 0.95(0.68, 1.32) |
| Q4 | 1.48(1.10,1.99) | | 1.38(1.04,1.83) | 1.45(1.03,2.03) | 1.48(1.10,2.02) | 1.45(1.07, 1.97) |
| P for trend | <0.01 | | 0.04 | 0.01 | 0.01 | 0.01 |
| **Without diabetes** |  |  |  |  |  |  |
| Continues | 0.99(0.94,1.04) | | 0.99(0.94,1.05) | 0.97(0.91,1.03) | 1.03(0.97,1.10) | 1.00(0.94, 1.06) |
| Quantiles |  | |  |  |  |  |
| Q1 | Ref | | Ref | Ref | Ref | Ref |
| Q2 | 0.91(0.84,0.99) | | 0.90(0.82,0.97) | 0.89(0.81,0.97) | 0.92(0.85,1.01) | 0.91(0.83, 0.99) |
| Q3 | 0.94(0.87,1.02) | | 0.94(0.86,1.01) | 0.92(0.84,1.01) | 0.98(0.90,1.07) | 0.95(0.87, 1.03) |
| Q4 | 0.95(0.87,1.03) | | 0.95(0.87,1.03) | 0.93(0.85,1.03) | 1.01(0.91,1.10) | 0.96(0.88, 1.05) |
| P for trend | 0.55 | | 0.58 | 0.39 | 0.46 | 0.50 |

*Note: Cox proportional hazards models were used to assess the relationship between the TyG index – measured both as a 1-unit increment and in quantiles – and sepsis-related mortality risk. Model 1 was adjusted for age, sex, and race; Model 2 additionally included the TDI, MET, diet score, tobacco/alcohol consumption, education level, employment status, and BMI. Other covariates included DBP, SBP, LDL, CRP, HDL, and CKD history*

*Abbreviations: TyG, triglyceride-glucose index; SD, standard deviation; TDI, Townsend Deprivation Index; MET, metabolic equivalent of task; BMI, body mass index; HR, hazard ratio; CI, confidence interval; Ref, reference.*

**Supplementary Figure 1. The flow chat of present study**

**
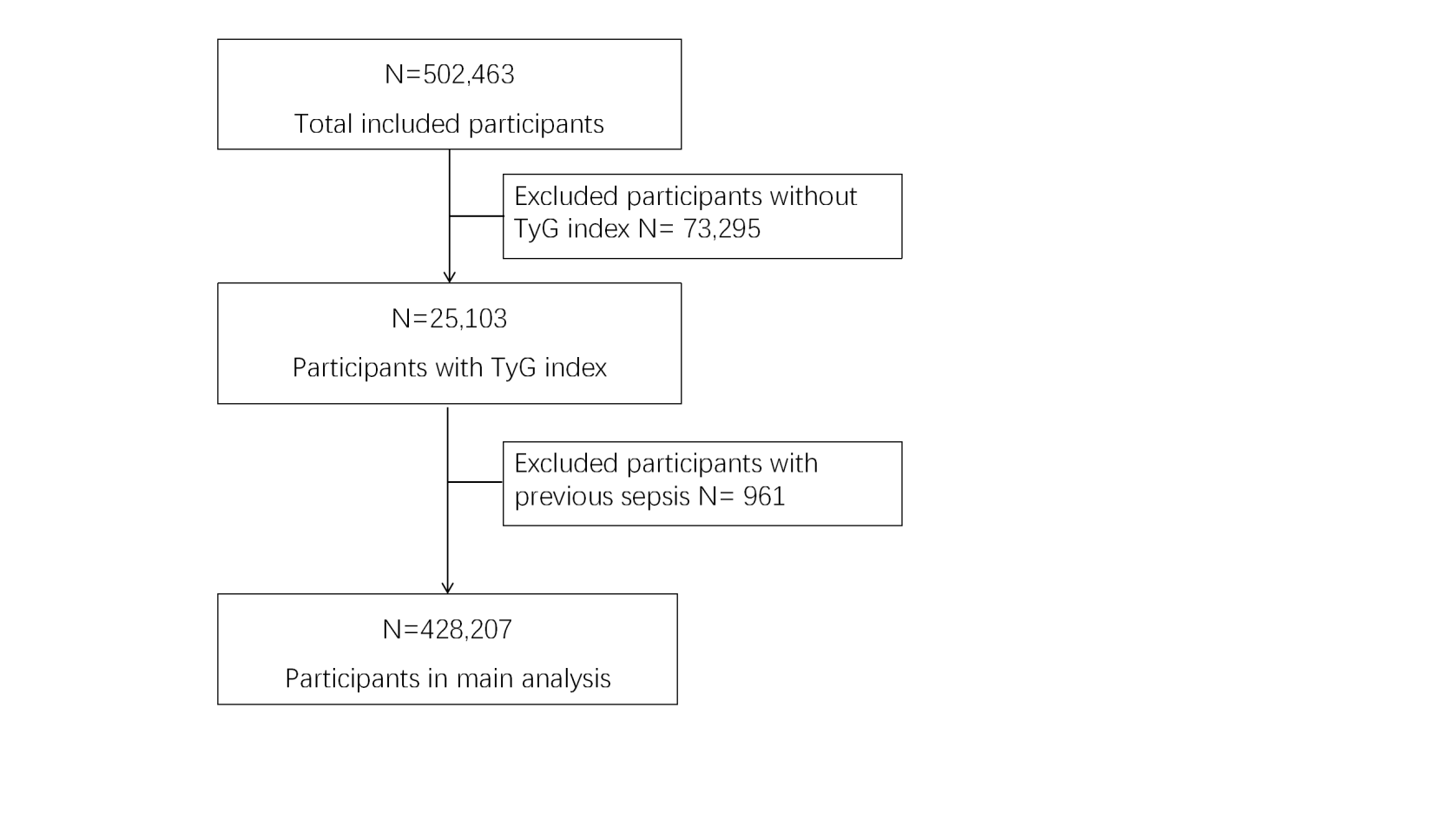
**
